# RNA methylation sequencing shows different gene expression signatures for response to azacytidine therapy in high‐grade myelodysplastic syndromes

**DOI:** 10.1111/jcmm.70078

**Published:** 2024-09-27

**Authors:** Diana Gulei, Vlad Moisoiu, David Kegyes, Rares Drula, Sabina Iluta, Adrian Bogdan Tigu, Madalina Nistor, Ciprian Jitaru, Anamaria Bancos, Petra Rotariu, Corina Popovici, Delia Dima, Radu Tomai, Ioana Rus, Catalin Constantinescu, Raluca Munteanu, Diana Cenariu, Ugur Sezerman, Mihnea Zdrenghea, Jaroslav Cermak, Hermann Einsele, Gabriel Ghiaur, Ciprian Tomuleasa

**Affiliations:** ^1^ Medfuture Research Center for Advanced Medicine Iuliu Hatieganu University of Medicine and Pharmacy Cluj‐Napoca Romania; ^2^ University Hospital and University of Zurich Zurich Switzerland; ^3^ Department of Hematology Iuliu Hatieganu University of Medicine and Pharmacy Cluj Napoca Romania; ^4^ Department of Hematology Ion Chiricuta Clinical Cancer Center Cluj Napoca Romania; ^5^ Department of Experimental Therapeutics University of Texas MD Anderson Cancer Center Houston Texas USA; ^6^ Department of Oncology Bistrita Emergency Hospital Bistrita Romania; ^7^ Department of Biostatistics and Medical Informatics, School of Medicine Acibadem Mehmet Ali Aydinlar University Istanbul Turkey; ^8^ Laboratory of Anemias Institute of Hematology and Blood Transfusion Prague Czech Republic; ^9^ Department of Internal Medicine II, Hematology University Hospital Würzburg Würzburg Germany; ^10^ Department of Leukemia, Sidney Kimmel Cancer Center at Johns Hopkins Johns Hopkins University School of Medicine Baltimore Maryland USA

**Keywords:** myelodysplastic syndromes, prognosis, RNA methylation sequencing

## Abstract

Myelodysplastic syndromes (MDS) are myeloid malignancies with heterogeneous genotypes and phenotypes, characterized by ineffective haematopoiesis and a high risk of progression towards acute myeloid leukaemia (AML). Prognosis for patients treated with hypomethylating agents (HMAs), as is azacytidine, the main drug used as frontline therapy for MDS is mostly based on cytogenetics and next generation sequencing (NGS) of the initial myeloid clone. Although the critical influence of the epigenetic landscape upon cancer cells survival and development as well on tumour environment establishment is currently recognized and approached within current clinical practice in MDS, the heterogenous response of the patients to epigenetic therapy is suggesting a more complex mechanism of action, as is the case of RNA methylation. In this sense, the newly emerging field of epitranscriptomics could provide a more comprehensive perspective upon the modulation of gene expression in malignancies, as is the proof‐of‐concept of MDS. We initially did RNA methylation sequencing on MDS patients (*n* = 6) treated with azacytidine and compared responders with non‐responders. Afterwards, the genes identified were assessed in vitro and afterwards validated on a larger cohort of MDS patients treated with azacytidine (*n* = 58). Our data show that a more accurate prognosis could be based on analysing the methylome and thus we used methylation sequencing to differentially split high‐grade MDS patients with identical demographical and cytogenetic features, between azacytidine responders and non‐responders.

## 1 INTRODUCTION

MDS represent a heterogenous group of disorders of the haematopoietic stem cell which are described by pathologists to have dysplasia in the myeloid, megakaryocytic and/or erythroid progenitors, with the abnormal clone being a (pre)malignant clone that progressively represses normal haematopoiesis. Patients often have peripheral cytopenia, with the natural evolution of the disease ranging from indolent disease (low‐grade MDS) that may span for several years, to a more aggressive manifestation (high‐grade MDS), when most patients either progress to acute myeloid leukaemia (AML) or eventually succumb due to bone marrow failure.^1^

The incidence is often estimated to be lower than it actually might be in the general population due to the complexity of diagnosis and classification of MDS, with around 70% being low‐grade.^2^ Diagnosis and classification includes morphology, histopathology and cytogenetic analysis, with flow cytometry and next generation sequencing (NGS) being important in classifying MDS according to the International Prognostic Scoring System (IPSS).^3^, ^4^ IPSS‐R subdivides MDS patients into prognostic groups and aids management decisions. Whereas allogeneic haematopoietic stem cell transplantation being the only curative option for MDS, firstline treatment for high‐grade MDS is based on hypomethylating agents (HMAs) for more than 10 years, having proven to prolong survival when compared to other regimens.^5^ Still, prognosis for patients treated with HMAs, as is azacytidine, the main drug used as frontline therapy for MDS is mostly based on cytogenetics and NGS of the initial myeloid clone.^6^

Although the critical influence of the epigenetic landscape upon a cancer cell's survival and development; as well on tumour environment establishment is currently recognized and approached within haematology clinical practice, the heterogenous response of the patients to epigenetic therapy is suggesting a more complex mechanism of action. In this sense, the newly emerging field of epitranscriptomics could provide a more comprehensive perspective upon the modulation of gene expression in malignant pathologies. N^6^‐methyladenosine (m^6^A) is the most common non‐genetic alteration in mRNAs and has role in RNA metabolism but also in mediating aberrant gene expression profiles with effects on installation and development of diseases. RNA methylation is mediated by m^6^A methyltransferases, removed by demethylases and identified by m^6^A binding proteins, all of them recognized as ‘writers’, ‘erasers’ and ‘readers’, respectively. The mechanism of RNA methylation in solid and haematological tumours is in its early years, without comprehensive clarification, and whatever this mechanism can surpass epigenetic changes is unknown. haematological patients that are non‐responders to demethylating agents could be characterized by a dominant RNA methylation profile that favours the cancer phenotype or could present compensatory feedback mechanisms at the level of RNA to impede the modifications from the DNA induced by epigenetic therapy.^7^, ^8^ Wide screening of different markers from the RNA methylation machinery could predict the eligible patients for epigenetic treatment, while analysis of the molecular background of the non‐responder patients could offer new insights into the mechanisms of dynamic RNA methylation in cancer. This is the first report that assess the role of RNA methylation in acquiring resistance to hypomethylating agents, but the data in the field is still scarce and further research in the field is required.

Real‐world data reveal that a substantial portion of patients with MDS do not respond to epigenetic therapy with hypomethylating agents, as is azacytidine. The cellular and molecular reasons for this resistance is largely unknown.

Our hypothesis is that a more accurate prognosis could be based on analysing the methylome and thus we used methylation sequencing to differentially split high‐grade MDS patients with identical demographical and cytogenetic features, between azacytidine responders and non‐responders.

## 2 MATERIALS AND METHODS

### 2.1 Initial screening MDS patient cohort

Six initial patients (*n* = 6) were enrolled in the current study, after having signed the informed consent forms. All clinic‐demographical characteristics were similar, all having the same age range (60–65 years old), all with a diagnosis of MDS, IPSS intermediate‐2 grade, with normal karyotype. We compared patients that had a haematological response to azacytidine therapy (*n* = 3), that have received at least 6 cycles of therapy, and afterwards lost the response and evolved towards AML to patients (*n* = 3) that maintained a long‐term haematological response to azacytidine therapy, achieving long‐term haematological response.

### 2.2 Total RNA fragmentation and m^6^A immunoprecipitation

Total RNA integrity was measured by Qubit RNA HS Assay and respectively Agilent 2100 Bioanalyzer (Agilent Technology). 10 μg of total RNA from each patient sample was afterwards fragmented by using a 10X RNA Fragmentation Buffer (Invitrogen) following incubation in a preheated thermal cycler at 70°C for 10 min. Fragmented RNA was then pelleted by ethanol precipitation. Both protein A and protein G magnetic beads were then washed twice using IP buffer (150 mM NaCl, 10 mM Tris–HCl pH 7.5, 0.1% IGEPAL CA‐630 in nuclease‐free H_2_O) and then incubated along with 5 μg m^6^A antibody (Synaptic Systems) for 2 h at 4°C. After being washed twice with the IP buffer, the antibody‐beads complexes were later resuspended in 500 μl of the IP reaction mixture, which included fragmented total RNA, before incubation at 4°C for 4 h. The immunoprecipitated m^6^A RNA with protein A/G magnetic beads was then washed 3 times using IP buffer for 10 min each at 4°C. Finally, the beads complexes were resuspended in 100 μl of m^6^A competitive elution buffer with continuous shaking for 1 h at 4°C. The supernatant that contains the eluted m^6^A RNA was later collected in another new tube and then purified using phenol:chloroform:isoamyl alcohol (125:24:1).

### 2.3 Library construction

The libraries that used the eluted RNA were constructed using the SMARTer Stranded Total RNA‐Seq Kit version 2 (Takara/Clontech). The eluted m^6^A RNA and input RNA were conducted directly first‐strand cDNA synthesis without additional fragmentation. The libraries for IP RNA were amplified by using PCR for less than 16 cycles, with input libraries being less than 12 cycles. All libraries were afterwards analysed by an Agilent 2100 Bioanalyzer (Agilent Technologies) and then quantified by real time PCR, before sequencing was performed.

### 2.4 Raw data filtration and sequence alignment

Low quality bases were trimmed using trimmomatic (version: 0.38). For this protocol, the options and parameters used for filtering the raw reads were as follows: ILLUMINACLIP:MeRIP‐PE.fa:2:30:10:1:true SLIDINGWINDOW:30:15 AVGQUAL:15 LEADING:15 TRAILING:15 MINLEN:3. Clean reads were afterwards mapped to Homo_sapiens.GRCh38.dna.toplevel.fa(Homo sapiens) genome (hg38) by using hisat2 (version:2.1.0) with the default parameters. The alignment file(SAM) was then transformed to BAM file and filtered according to the following steps: (1) only keep unique properly aligned reads; (2) remove reads with low MAPQ (<30). (3) remove reads mapped to blacklist regions.

### 2.5 Peak calling and differential peak detection

Peaks and detection of differential peaks from filtered alignment files with parameters that ‘WINDOW_WIDTH=200 SLIDING_STEP=30 FRAGMENT_LENGTH=150 DIFF_PEAK_ABS_FOLD_CHANGE = 2‐FOLD_ENRICHMENT=2’ were called by using R package exomePeak (version: 2.1.2). The annotation file used to annotate the peaks was downloaded from UCSC. Only peaks with FDR <0.05 and foldchange > = 2 were identified as being significantly differential peaks.

### 2.6 Gene expression calculation

Gene expression calculation was performed with by StringTie software, according to the default parameters. Gene expression profiling was based according to the number of reads. TPM (Transcripts Per Million mapped reads) values estimated the expressed values and transcript levels. DESeq2 identified differentially expressed genes (DEGs). Genes with an adjusted *p* value (padj) <0.05 and abs (log2(fold change)) >1 were assigned as DEGs. GO and KEGG enrichment analysis was performed by cluster profiler. KEGG (Kyoto Encyclopedia of Genes and Genomes) (http://www.kegg.jp/) is a collection of databases that deals with genomes, biological pathways, diseases, drugs, as well as chemical substances. This was used for bioinformatics analysis. Gene ontology (GO) (http://www.geneontology.org), a major bioinformatics initiative unifies the representation of gene and gene product attributes across all species.

### 2.7 Assessment of the RNA methylation sequencing results in vitro

The MDS‐L cell line was cultured in RPMI‐1640 medium supplemented with 10% fetal bovine serum 1% glutamine and 1% penicillin–streptomycin. The culture medium and all the supplements were acquired from Gibco. During the experiments, the cells were maintained in a 5% CO_2_‐filled, sterile, humidified chamber. MDS‐L cells were cultured in 96‐well plates and treated in triplicate with 10 increasing concentrations of azacytidine, ranging from 0 to 100 micromolar in order to determine the half‐maximal inhibitory concentration (IC_50_). Every 24 h, azacytidine was added to the culture media. After 72 h of treatment, live cells were counted using the NanoEntek EVE automated cell counter and 0.4% trypan blue (purchased from EVE Countess). In subsequent studies, MDS‐L cells were exposed to inhibitory concentration 25 (IC25 = 53 micromolar), which was established by means of prior cell viability tests. Every 24 h, azacytidine was added to the culture medium. Following a 72‐h treatment period, treated and control cells were collected, washed and kept in 800 microliters of Trireagent.

### 2.8 Validation MDS patient cohort

The bone marrow aspirate from patients diagnosed with MDS (*n* = 58) was collected on K2EDTA coated tubes and processed immediately to avoid sample denaturation. The Mononuclear cells were isolated by Ficoll separation at 650×g for 30 min with slow acceleration and deceleration. The Cells were further washed with PBS1X and stored in 800 μL of Trireagent (Thermo Scientific, Waltham, MA, USA) until further use.

The RNA was extracted from cells and patient samples that were kept in Trireagent at −80°C. The extraction protocol started by mixing 160 μL of chloroform with 800 μL of sample in Trireagent, followed by a mixing step and an incubation at room temperature for 5 min. The samples were centrifuged at 10.000×g at 4°C for 20 min, then the aqueous phase (clear part of the sample) was transferred in another sterile 1.5 mL tube and mixed with 500 μL of 2‐propanol, vortexed and incubated at room temperature for 10 min. The samples were centrifuged at 13.000×g for 15 min at 4°C, and the supernatant was carefully removed without disturbing the RNA pellet. On top of the pellet, 1 mL of pure Ethanol was carefully poured, and the samples were centrifuged at 17800×g for 10 min at 4°C and the supernatant was carefully removed. After 15 min drying step, the pellet was dissolved in ultrapure water (50 μL) and kept on ice until reading the RNA samples using the NanoDrop2000 system (Waltham, MA, USA). All reagents were purchased from Thermo Scientific (Waltham, MA, USA). The resulted RNA was further processed for cDNA synthesis, starting with a DNAse treatment step, according to the TURBO DNAse Kit protocol (Thermo Scientific, Waltham, MA, USA). The Pure RNA resulted after the treatment was measured using the NanoDrop2000 system, and the same amount of RNA was used for cDNA synthesis step. The cDNA synthesis was performed according to the protocol for High‐Capacity cDNA Reverse Transcription Kit (Thermo Scientific, Waltham, MA, USA). The cDNA was further used for RT‐PCR assays.

The RT PCR analysis was performed using the SyBr Green PCR Master Mix 2X (Thermo Scientific, Waltham, MA, USA), at a final volume of 20 μL of mix. The reaction was analysed using the Step One Plus RT PCR machine (Thermo Scientific, Waltham, MA, USA), in 96 well plates. The results were calculated as relative gene expression (2^‐DCT) comparing with the control samples. The statistical analysis was performed using GraphPad Prism 8 software.

## 3 RESULTS

### 3.1 Functional enrichment analysis of differential peak‐associated gene

**FIGURE 1:**
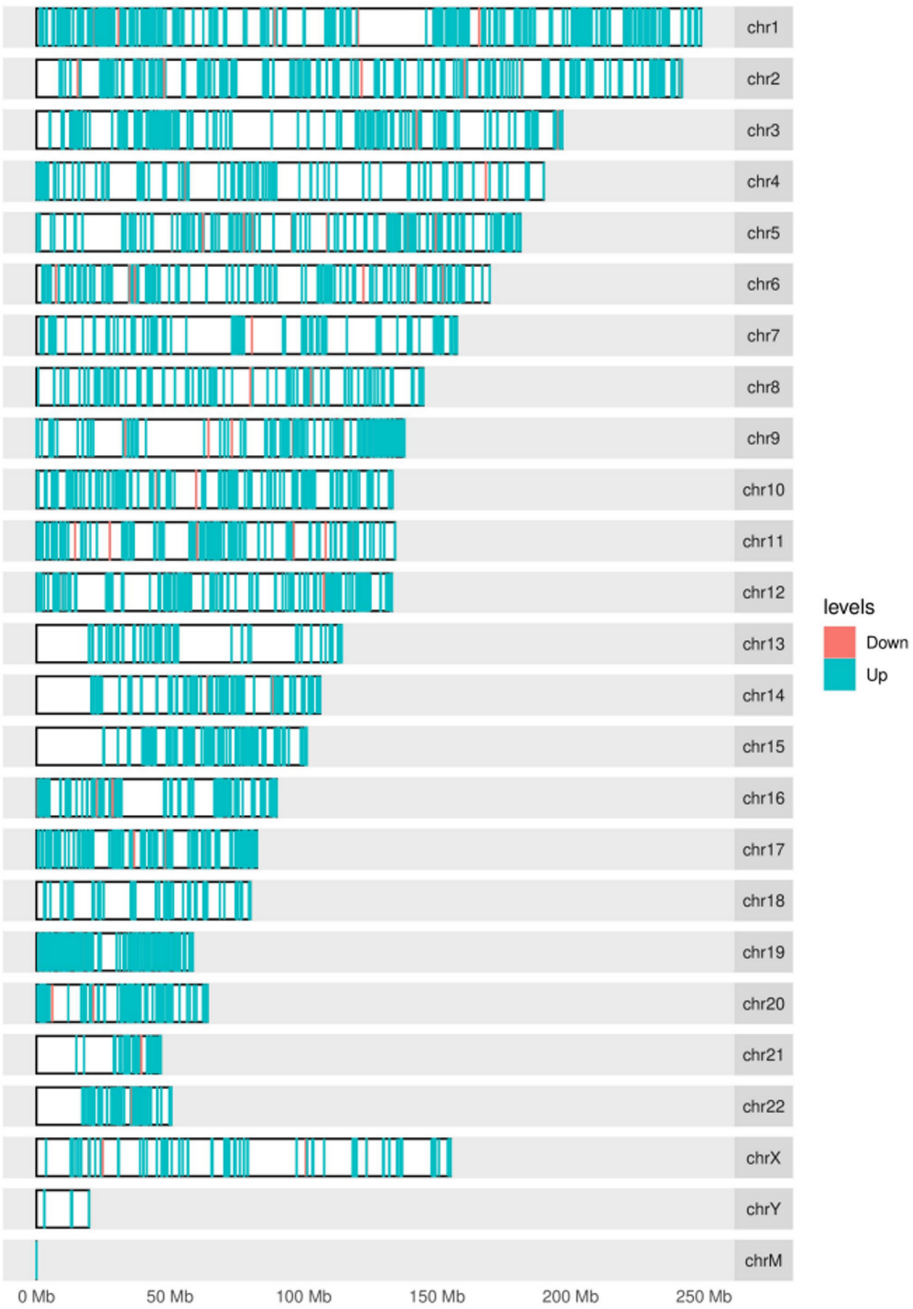
Distribution of differential peaks on chromosomes, comparing responders versus non‐responders. Most differential peaks are upregulated when comparing responders with non‐responders.

**FIGURE 2:**
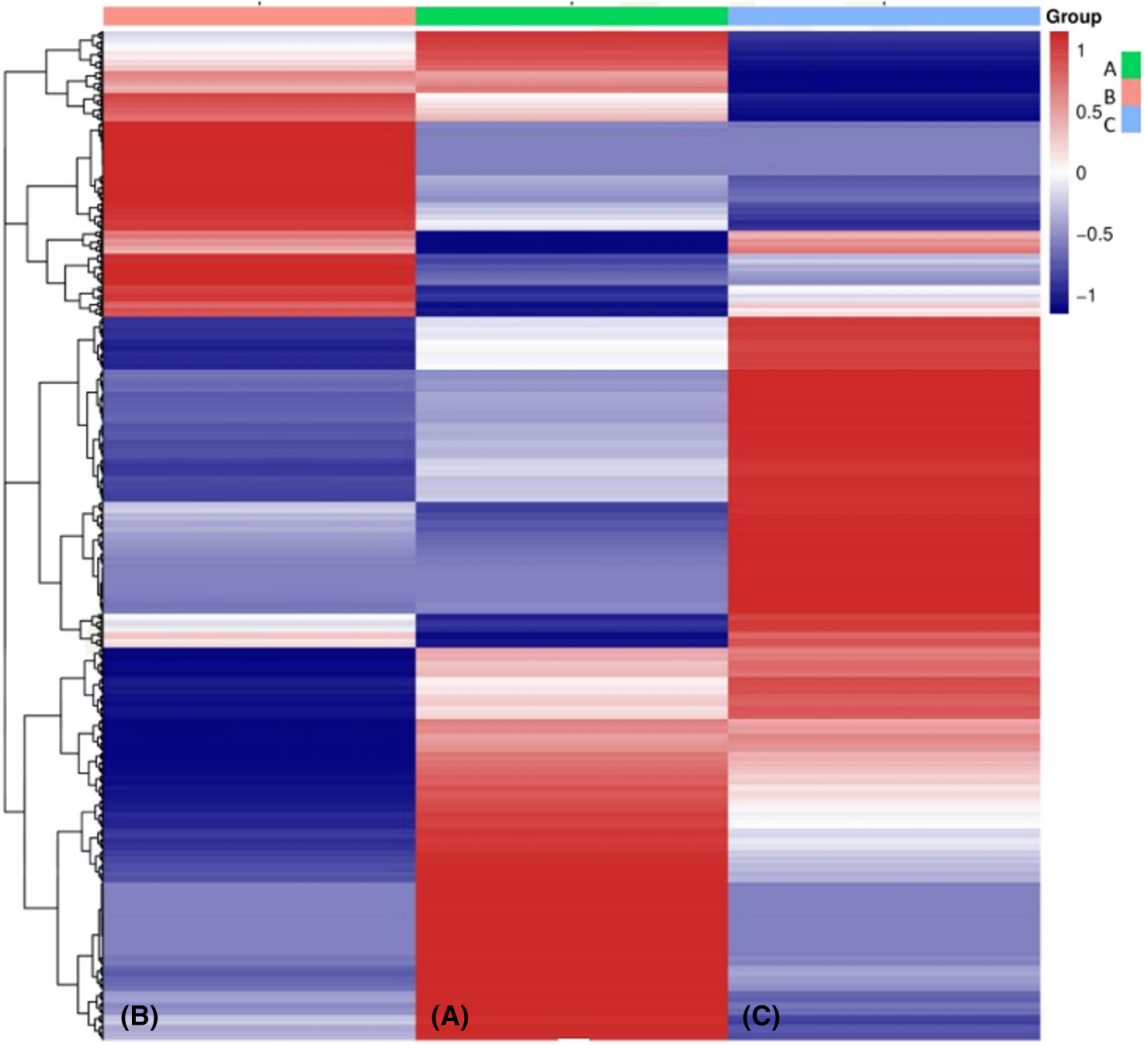
Heat map of differentially expressed genes (DEGs). (A) represents the patients that were treated with azacitidine, achieved remission and maintained the remission. (B) represents the patients that initially achieved a remission but relapsed and the disease progressed to AML. (C) represents the patients that were refractory from the beginning to azacytidine therapy and progressed to AML.

**FIGURE 3:**
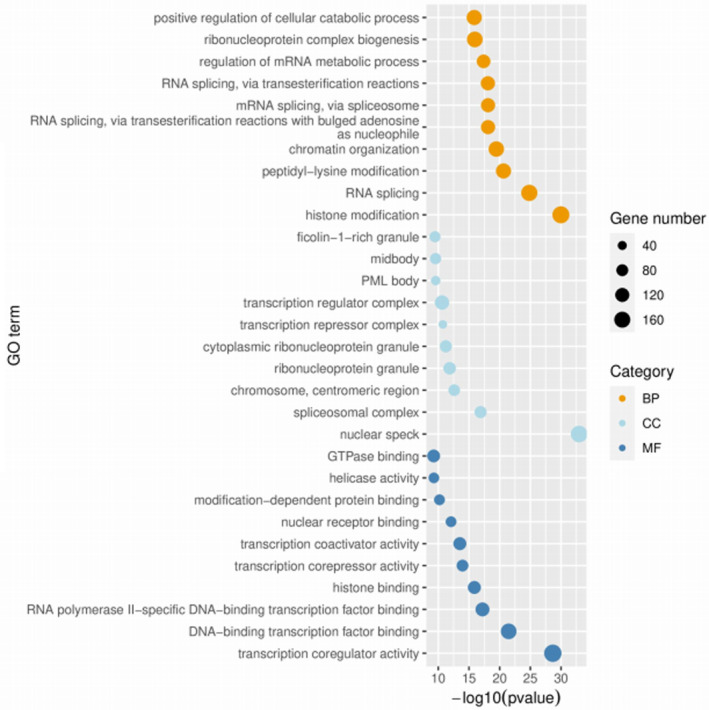
Scatter plot of enriched TOP30 GO term.

The differential peak distribution on the chromosomes of the regions that are methylated most and, respectively, least in MDS patients that responded to azacytidine monotherapy is shown in Figure 1. The project aimed to maintain and develop its own controlled vocabulary of gene and gene product attributes, as well as annotate genes and gene products, thus assimilating and disseminating annotation data. This allows a tool for the easy access to all of the data generated by the project and thus enables a functional interpretation of experimental data by using GO. One eloquent example is enrichment analysis. Functional enrichment was performed in three categories of GO terms: Biological process (BP), molecular function (MC) and cellular component (CC). Figure 2 depicts the genes differentially expressed (DEGs) between responders and non‐responders, along with their function (Figure 3).

### 3.2 Correlation between differentially expressed genes and peaks

The four‐quadrant graphic of m^6^A‐related genes and DEGs in responders and non‐responders is depicted in Table 1. According to Table 1, a potential gene with clinical impact in RNA methylation following therapy with azacytidine for MDS might be ENSG00000000938, or SRC2, a FGR proto‐oncogene, not previously reported to play a role in RNA methylation of leukemogenesis.

**TABLE 1:** m^6^A‐related genes and EDGs (responders vs. non‐responders).

Gene	m_FC	m_FDR	m_sig	g_FC	g_FDR	col
ENSG00000000460	2.01	−12	Yes	−0.39150145	0.820533482	Grey
ENSG00000000460	1.12	−1.92	Yes	−0.39150145	0.820533482	Grey
ENSG00000000938	2.29	−6.4	Yes	2.175587417	7.68E‐21	Red
ENSG00000001167	1.25	−1.99	Yes	−0.191253868	1	Grey
ENSG00000001497	0.653	−2.09	Yes	−0.641926715	0.038013886	Orange
ENSG00000001497	1.76	−12	Yes	−0.641926715	0.038013886	Orange
ENSG00000001561	0.799	−2.12	Yes	−0.065072858	1	Grey

Abbreviations: col, color in four‐quadrant graph; g_FC, log2(fold change) of diff gene; g_FDR, log10(FDR) of diff gene; Gene, gene symbol; m_FC, log2(fold change) of diff m^6^A; m_FDR, log10(FDR) of diff m^6^A.

### 3.3 In vitro assessment on the effect of azacytidine on MDS

**FIGURES 4:**
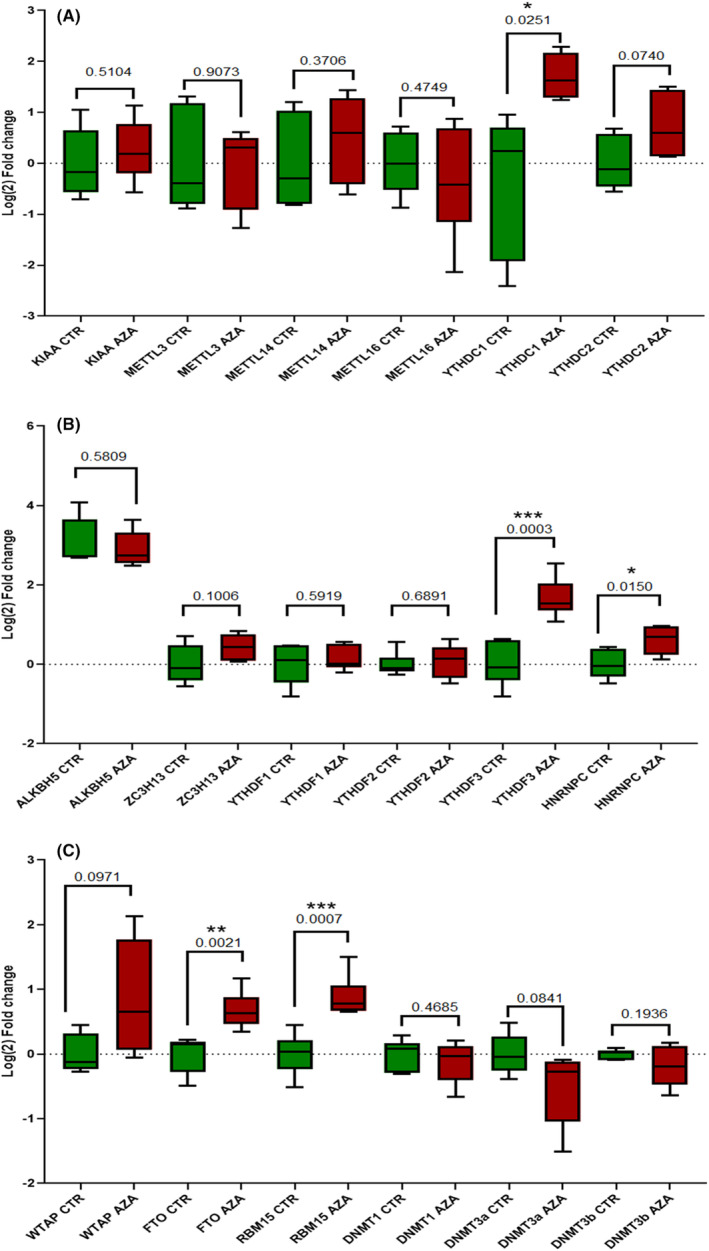
(A–C) In vitro assessment of the differentially expressed genes that were upregulated in responders versus non‐responders following RNA methylation sequencing on the initial cohort. The in vitro tests used an MDS cell line treated with azacytidine. *p < 0.05; **p < 00.01; ***p < 0.001

For the MDS‐L cell line, the gene expression indicated five genes with statistically significant changes in their relative expression. The results are depicted in Figure 4A–C. The analysis was performed using a *t*‐test with Welch's correction. The results are expressed as *p < 0.05, **p < 0.01 and *** for *p* < 0.001, where *n* = 3 (number of replicates).

### 3.4 Validation of the in vitro data using an MDS patient cohort

Validation of the data obtained in vitro was carried out using an extended MDS patient cohort. The Samples were divided into two groups, the untreated ones (at diagnosis, where *n* = 58) and the treated ones (after at least 1 cycle of Aza, where *n* = 13). The patient characteristics of the patient cohort is shown in Table 2.

**TABLE 2:** MDS patient cohort characteristics.

Characteristic	*N* ^a^=58
Age	68 (9)
WBC	11 (16)
Hb	9.28 (2.36)
Plt	87 (86)
Sex	
F	22/58 (38%)
M	36/58 (62%)

^a^ Mean (SD); *n*/*N* (%).

**FIGURE 5:**
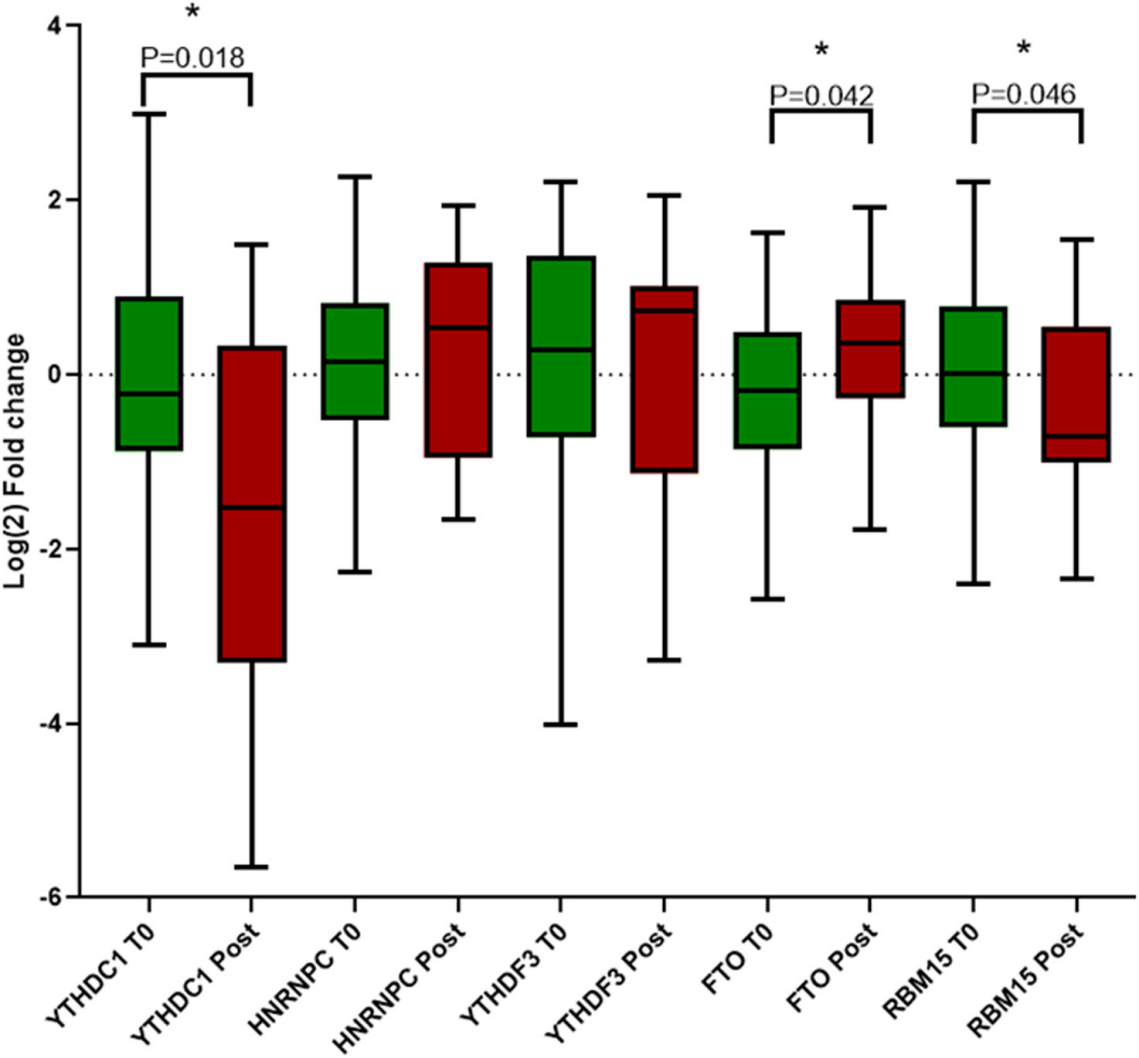
RT PCR analysis on MDS samples from patients of the validation cohort. T0—patient at diagnosis. Post—patients after azacytidine treatment. *p < 0.05.

For the five genes that displayed significant changes between the experimental groups in vitro, we have performed the RT‐PCR analysis on samples from patients (Figure 5).

## 4 DISCUSSION

For high‐grade MDS, in the United States and Europe, the only curative therapeutic option is an allogeneic stem cell transplantation. Should a transplant not be a foreseeable option for the patient, as they are not eligible for intensive chemotherapy, HMAs represent the frontline standard‐of‐care. Azacytidine is mostly used, and many attempts have been used so far to increase the efficacy by adding novel drugs, but with limited success so far. Using azacytidine is often associated with unpredictable results in the clinic, with patients that apparently have similar demographic, genetic and haematological characteristics having different outcomes. Some achieve long‐term haematological remission, whereas other progress to AML being afterwards refractory to induction chemotherapy. This clinical observation may be the direct consequence of methylation of both DNA, for which azacytidine was proven to be effective, as well as a direct of RNA methylation. RNA methylation following therapy with azacytidine for MDS was investigated in the current manuscript, aiming to differentially separate responders from non‐responders.

**FIGURE 6:**
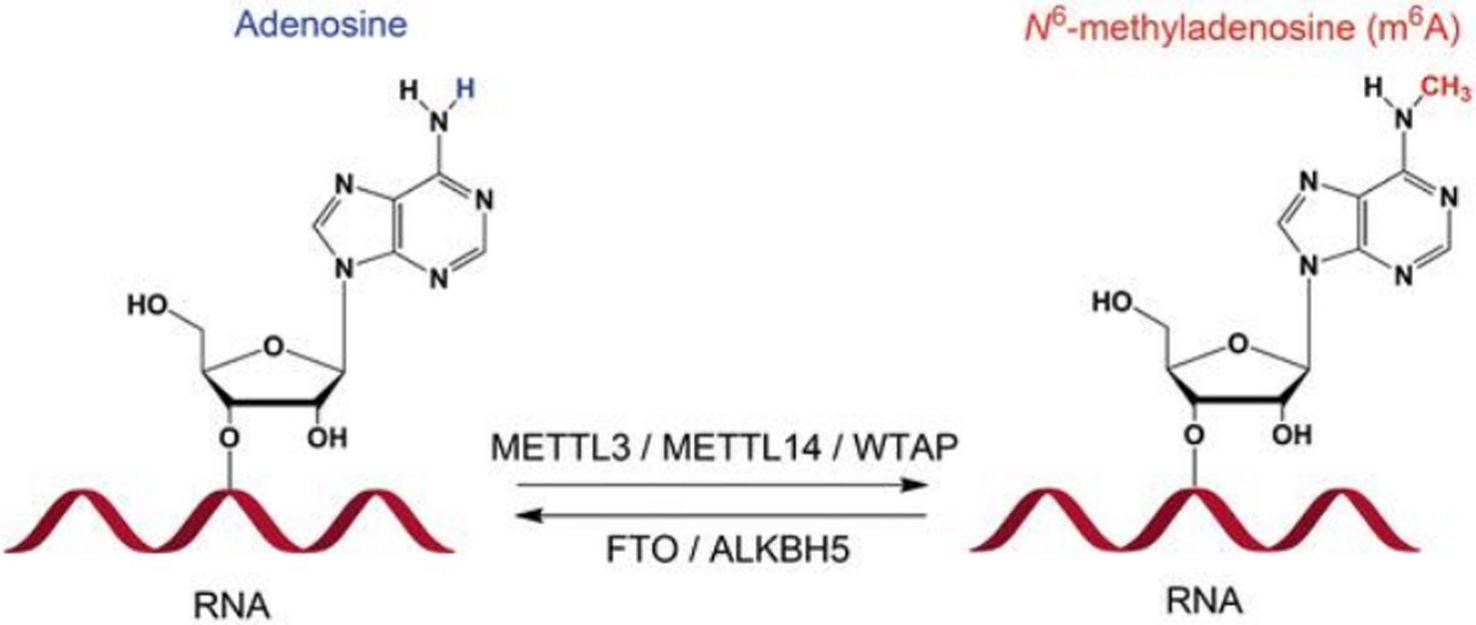
RNA m^6^A methylation is a post‐transcriptional modification that occurs at the nitrogen‐6 position of adenine. This dynamically reversible modification is installed, removed and recognized by methyltransferases, demethylases and readers, respectively. This modification has been found in most eukaryotic RNA species.

**FIGURE 7:**
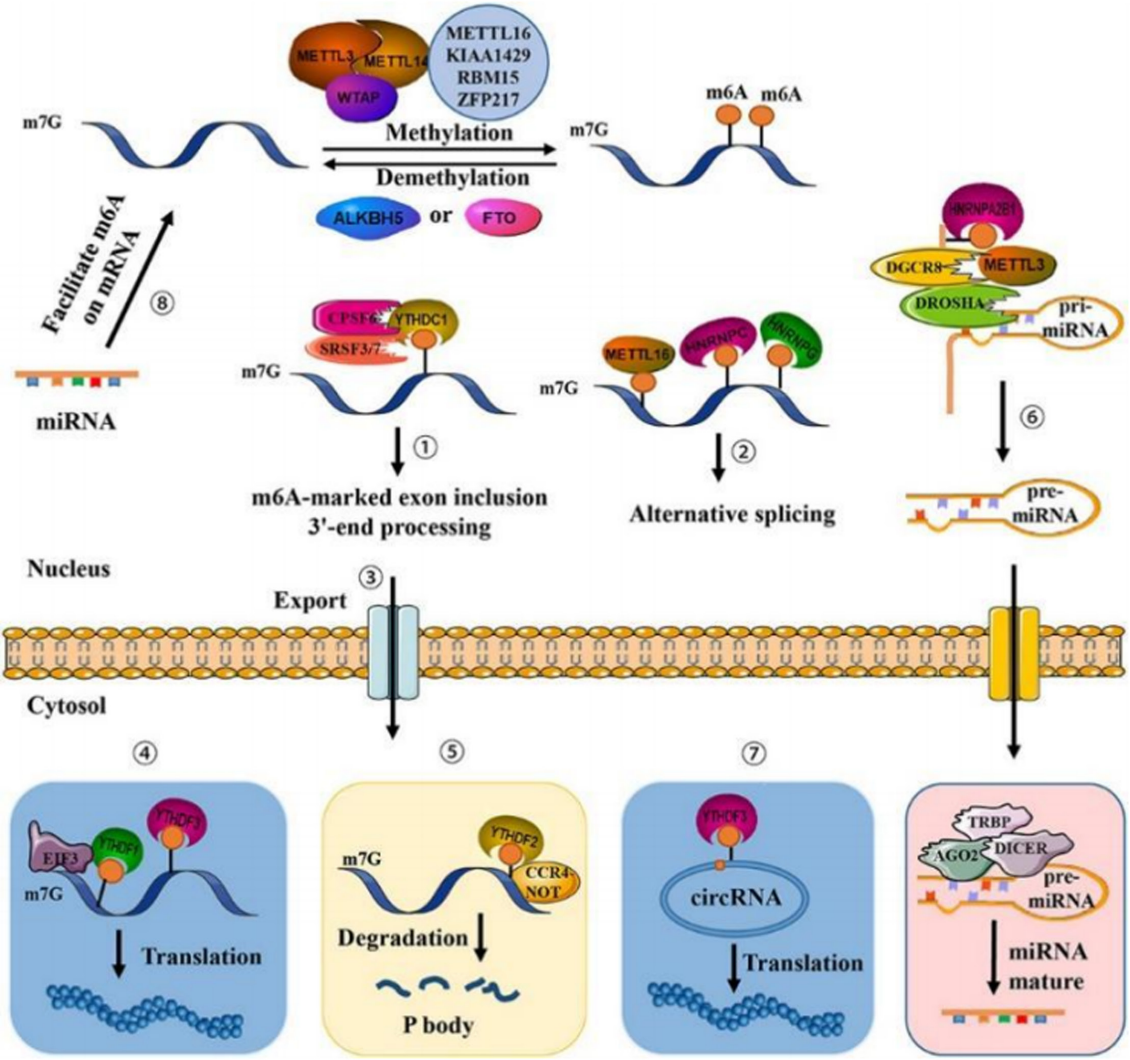
m^6^A as an mRNA modification that is abundant in some viruses and nearly all eukaryotes. A variety of cytopathologic processes involving nuclear RNA export, splicing, mRNA stability, circRNA translation, miRNA biogenesis and lncRNA metabolism have recently been linked to aberrant levels of m^6^A. In addition, m^6^A modification has been associated with numerous physiological and pathological phenomena, including obesity, immunoregulation, yeast meiosis, plant development and carcinogenesis.

RNA m^6^A methylation is a post‐transcriptional change that appears at the nitrogen‐6‐position of adenine. This modification is dynamically reversible and is installed, removed and recognized by methyltransferases, demethylases and readers, respectively. RNA m^6^A methylation is reported in most of the eukaryotic mRNA, tRNA and other non‐coding RNAs. m^6^A is shown to be present in 0.1%–0.4% of all the adenosines in global cellular RNAs and accounts for 50% of all methylated ribonucleotides, as proven by Figure 6. The RNA methyltransferase complex comprises the methyltransferase‐like 3 (METTL3)/METTL14 core subunits, as well as other cofactors and is responsible for the deposition of m^6^A on mRNA. METTL3 is the catalytic subunit and METTL14 is crucial to target recognition. Wilms' tumour 1‐associating protein (WTAP) regulates the recruitment of the m^6^A methyltransferase complex to mRNA targets. m^6^A is an mRNA modification identified in some viruses, but in nearly all eukaryotes. Various cytopathological processes involve nuclear RNA export, splicing, miRNA biogenesis and lncRNA metabolism, as well as mRNA stability or circRNA translation and have recently been linked to aberrant levels of m^6^A. Additionally, m^6^A modification is linked to numerous physiological and pathological phenomena, including human metabolism, immunoregulation, development, carcinogenesis and resistance to therapy, as is the case of the present data (Figure 7).

DNA methylation signature has been shown to be different between MDS patients that respond to HMAs therapy.^9^, ^10^ Still, azacytidine de‐methylates DNA, but not RNA. Thus, RNA methylation was not previously reported to play a role in differentiating MDS patients between responders and non‐responders. As azacytidine has little or no effect on RNA methylation, genes differentially expressed between responders and non‐responders might potentially play a role in predicting MDS response to therapy. One potential such gene is the SRC2 gene, not previously reported to be linked to methylation status in cancer, but with an interesting potential in MDS and leukaemia biology. This hypothesis is far from being new, as proven by the work of Cheng et al,^11^ in leukaemia. In all malignancies, as well as in pre‐malignancies, as is the case of MDS, resistance to treatment is often leading to a dismal prognosis and patient death. Resistance to treatment, including chemotherapy or targeted therapy, can be influenced by RNA epigenetics and chromatin structure. Cheng et al have shown that for malignant cells, RNA 5‐methylcytosine and RNA: m^5^C methyltransferases mediate chromatin structures, leading to resistance to epigenetic treatment. Our manuscript comes to support this data, in a less aggressive myeloid disorder, as is MDS. Still, once this biological phenomenon develops, these patients lose treatment response and evolve towards AML, often to dismal outcomes.

Still, a large cohort is further needed to compared responders with non‐responders, as well as a potential biological process of ‘gain of RNA methylation’ following azacytidine therapy, as a biological background of gain of resistance to treatment and AML progression.

## AUTHOR CONTRIBUTIONS

**Diana Gulei:** Conceptualization (equal); investigation (equal). **Vlad Moisoiu:** Formal analysis (equal). **David Kegyes:** Formal analysis (equal). **Rares Drula:** Investigation (equal). **Sabina Iluta:** Investigation (equal). **Adrian Bogdan Tigu:** Investigation (equal). **Madalina Nistor:** Investigation (equal); visualization (equal). **Ciprian Jitaru:** Investigation (equal). **Anamaria Bancos:** Investigation (equal). **Petra Rotariu:** Investigation (equal). **Corina Popovici:** Methodology (equal); visualization (equal). **Delia Dima:** Investigation (equal); methodology (equal). **Radu Tomai:** Methodology (equal). **Ioana Rus:** Methodology (equal). **Catalin Constantinescu:** Investigation (equal). **Raluca Munteanu:** Investigation (equal). **Diana Cenariu:** Investigation (equal). **Ugur Sezerman:** Validation (equal); visualization (equal). **Mihnea Zdrenghea:** Investigation (equal); methodology (equal). **Jaroslav Cermak:** Formal analysis (equal); investigation (equal); methodology (equal). **Hermann Einsele:** Investigation (equal); methodology (equal). **Gabriel Ghiaur:** Conceptualization (equal); formal analysis (equal); project administration (equal). **Ciprian Tomuleasa:** Conceptualization (equal); funding acquisition (equal); project administration (equal).

## CONFLICT OF INTEREST STATEMENT

No potential conflict of interest is reported.

## Data Availability

The data that support the findings of the study are available from the corresponding author upon reasonable request.
